# Potential impacts of climate change on the geographic distribution of *Achillea eriophora* DC., a medicinal species endemic to Iran in southwestern Asia

**DOI:** 10.1002/ece3.11241

**Published:** 2024-04-25

**Authors:** Fariba Noedoost, Maryam Behroozian, Sahar Karami, Mohammad Reza Joharchi

**Affiliations:** ^1^ Department of Biology, Faculty of Science Behbahan Khatam Alanbia University of Technology Behbahan Khuzestan Iran; ^2^ Herbarium FUMH Ferdowsi University of Mashhad Mashhad Iran; ^3^ Quantitative Plant Ecology and Biodiversity Research Lab, Department of Biology, Faculty of Science Ferdowsi University of Mashhad Mashhad Iran

**Keywords:** *Achillea eriophora*, climate change, endemic, habitat suitability, MaxEnt model, Medicinal species

## Abstract

Climate change is considered to rank among the most important global issues affecting species' geographic distributions and biodiversity. Understanding effects of climate change on species can enhance conservation efficacy. In this study, we applied ecological niche modeling (ENM) using maximum entropy (MaxEnt) approaches to predict the potential geographic distribution of *Achillea eriophora* DC., a medicinal plant species to Iran in southwestern Asia, under current and future climate scenarios. We evaluated potential distributional areas of the species, under two shared socioeconomic pathways (SSP2‐4.5 and SSP5‐8.5) for the period 2041–2060. Most current potential suitable areas were identified for *A. eriophora* in montane regions. Our results anticipated that the potential distribution of *A. eriophora* will expand geographically toward higher elevations and northward. However, the species is expected to experience relatively high losses of suitability in its actual habitats under future climate scenarios. Consequently, we recommend regional‐to‐national conservation action plans for *A. eriophora* in its natural habitats.

## 1 INTRODUCTION

*Achillea eriophora* DC. (Asteraceae) is a medicinal plant species endemic to Irano‐Turanian and Sahara‐Sindian floristic regions in Iran (Mozaffarian, 2008; Rechinger, 1986). This species grows at the elevations of 700–2000 m, in arid and semi‐arid regions of Fars, Hormozgan, South Khorassan, Khuzestan, Sistan, Baluchestan, and Yazd provinces (Mozaffarian, 2008; Rechinger, 1986). *Achillea eriophora* is a perennial species with woody branches at the base, covered with dense woolly hairs. It is used in traditional medicine in Iran to treat digestive problems, diarrhea, fever, diabetes, bone pain, wounds, antipyretic, diuretic, carminative, and insect bite effects (Mehrabani et al., 2014; Sadat‐Hosseini et al., 2017). Considerable research has focused on the essential oils of *A. eriophora* in terms of its chemical constituents and biological activities (Etehadpour & Tavassolian, 2019; Ghani et al., 2011; Mottaghi et al., 2016). Biosynthesis of these secondary metabolites is influenced by various environmental factors (e.g., temperature, precipitation, and soil; Barra, 2009; Etehadpour & Tavassolian, 2019; Gudaitytė & Venskutonis, 2007; Moghaddam & Farhadi, 2015; Nemeth, 2005). For example, soil pH effects, chemical composition of essential oils (Barra, 2009), and essential oils of other species are affected by latitude and climate conditions (Azevedo et al., 2002; Hassiotis et al., 2014; Letchamo et al., 1995). The species has been categorized as of Least Concern (LC) by the International Union for the Conservation of Nature (IUCN) criteria (IUCN, 2011). However, it is illegally harvested in its habitats owing to its useful medicinal properties; environmental changes such as global climate change can be add future pressure on its conservation states (Behroozian, et al., 2020; Karami et al., 2022; Mohammadi et al., 2021, 2023).

An endemic species can be defined as a species with a restricted geographic range, sometimes related to a specific geographic area, reduced population size and adaptive ability, and limited dispersal capacities (Chichorro et al., 2019; Staude et al., 2020). Rare and threatened plants are more sensitive to environmental change and are at a higher risk of extinction owing to their narrow geographic distributions, low abundance, and highly specific habitat requirements (Behroozian et al., 2022; Coelho et al., 2020; Liao et al., 2023; Markham, 2014; Rejmánek, 2018). Hence, endemic species should be monitored carefully, and their conservation should be considered a universal priority (Foggi et al., 2015; Işik, 2011).

Climate change has considerably influenced the biological diversity and geographic species' distributions, especially rare and endemic species with narrow ranges (IPCC, 2014; Liao et al., 2023). The most common impacts of climate change on the distributions of species are that they shift, expand, or retract (Baumbach et al., 2021; Behroozian et al., 2020; Chaudhary et al., 2021; Khanal et al., 2022; Nabout et al., 2011; Yasuhara et al., 2020). Climate plays a crucial role in determining geographic distributions of plant species by shifting distributions or by adapting to changing conditions at broad scales (Behroozian et al., 2020; Erfanian et al., 2021; Ferrarini et al., 2019; Mousavi Kouhi et al., 2020; Parmesan, 2006; Schippers et al., 2011; Thuiller et al., 2005; Walther et al., 2002). When species are not able to adapt quickly enough to cope with these changes, they experience range loss, or may go extinct (Das, 2010; Lindzen, 1990; Sharma et al., 2020). Understanding climate change impacts is therefore crucial to addressing this global environmental challenge (Cavaliere, 2009).

Many studies have assessed the impact of climate change on potential geographic distributions of endemic species (Ardestani & Ghahfarrokhi, 2021; Erfanian, et al., 2021; Karami et al., 2022; Shaban et al., 2023; Wu et al., 2019). Despite the reported negative effects of climate change, warmer temperatures may benefit some medicinal and endemic species by permitting them to expand their ranges into currently unoccupied areas (Behroozian et al., 2020; Karami et al., 2022; Shaban et al., 2023). For example, Karami et al. (2022) found that climate change leads to an increase in the potential distribution of an endemic medicinal plant species, *Nepeta glomerulosa*, under future scenarios. Behroozian et al. (2020) suggested that climate change may be shifting the latitudinal distributions of an endemic plant species in mountains of Iran‐Turanian region, although suitability may have been reduced at local scales.

Ecological niche modelings (ENMs) are widely used in the fields of ecology, evolution, and conservation (Elith & Leathwick, 2009) to relate species occurrence data to environmental conditions to understand the relationship between species and their environments. ENMs also predicts the potential distribution of species under the present and specific environmental change scenarios. Maximum entropy approaches have been developed as a powerful method for modeling species and niches, even with limited occurrence data (Graham et al., 2008). These methods have been used widely to predict potential distributional areas of rare and endangered species, plan habitat conservation, and prioritize protected areas under changing climates (Condro et al., 2021; Qin et al., 2017; Yang et al., 2021).

In this study, we used ENM to assess the geographic potential of *Achillea eriophora*, a medicinal species in southwestern Asia, under current conditions and then transfer those models to the future under different scenarios. Our aims were (1) to identify the most significant environmental factors influencing the potential distribution of *A. eriophora* and (2) to evaluate the potential distribution of this species under various climate change scenarios. No studies have been conducted to consider the effects of climate change on the potential distribution of *Achillea eriophora* until now.

## 2 MATERIALS AND METHODS

### 2.1 Data collection

Primary occurrence records, including geographic coordinates for each record of the species, were obtained from the Global Biodiversity Information Facility (GBIF; https://gbif.org/) and SpeciesLink (http://splink.cria.org.br/). We used literature sources, including Flora Iranica (Rechinger, 1986), Flora of Iran (Mozaffarian, 2008), and Red Book of Iran. We also accessed literature and herbarium specimens in the collections of the Ferdowsi University of Mashhad Herbarium (FUMH). Occurrence records were entered into Microsoft Excel and saved in “.csv” format.

### 2.2 Environmental data

Climatic variables for the present and under two scenarios of future conditions (SSP2‐4.5 and SSP5‐8.5) were downloaded from WorldClim version 2.1 (a database of high spatial resolution global weather and climate data; https://www.worldclim.org/data/cmip6/cmip6_clim30s.html) at a spatial resolution of 30″ (about 1 km). Seven general circulation models (GCMs) were obtained for each scenario, including (1) Beijing Climate Center Climate System Model (BCC‐CSM2‐MR), (2) Australian Community Climate and Earth System Simulator Climate Model Version 2 (CCESS‐CM2), (3) Centro Euro‐Mediterraneo sui Cambiamenti Climatici—Earth System Model Version 2 (CMCC‐ESM2), (4) Goddard Institute for Space Studies Earth System Model (GISS‐E2‐1‐G), (5) Hadley Centre Global Environment Model in the Global Coupled Configuration 3.1 (HadGEM3‐GC31‐LL), (6) Institut Pierre‐Simon Laplace‐ Coupled Model Intercomparison Project (IPSL‐CM6A‐LR), and (7) Model for Interdisciplinary Research on Climate Version 6 (MIROC6) (Table S1).

### 2.3 Data preparation

**FIGURE 1:**
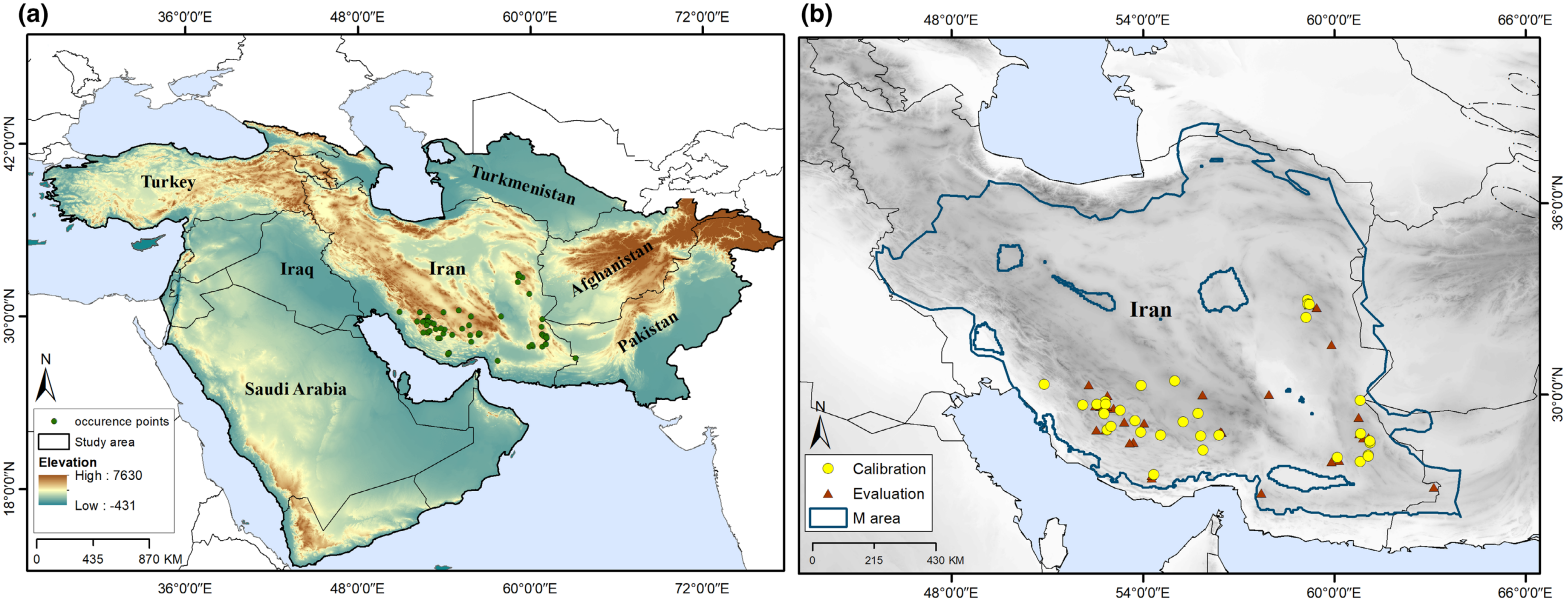
Study area and distribution of *Achillea eriophora*. (a) The distribution of *A. eriophora* in southern and eastern Iran. (b) Occurrence records of *A*. e*riophora* in a broader view, with the accessible area (**M**) for model calibration.

All occurrence records were mapped using ArcGIS 10.3 (geographical information system software; https://www.esri.com) for visualization and to check spatial accuracy. To avoid problems with spatial autocorrelation, we rarefied the occurrence data spatially based on a 1.5 km distance filter using the spThin library in R version 3.5.1 (Aiello‐Lammens et al., 2015). We divided the cleaned occurrence data into two equal parts at random to permit model calibration and evaluation (Figure 1b).

For bioclimatic variables, we excluded four of these variables (bio8, bio9, bio18, and bio19) from all analyses because they include known spatial artifacts (Escobar et al., 2014). Then, we calculated Pearson correlation coefficients among environmental variables to avoid using collinear variables in model calibration, in R version 3.5.1. One variable from each pair of the 15 climatic variables presenting high correlations (i.e., *r* > .8) was eliminated (Bemmels et al., 2016; Paulo et al., 2015). We applied the jackknife approach to select five sets of variables by removing the variable with the lowest independent contributions, creating sets of 6, 5, 4, and 2 variables for analysis.

We estimated a realistic calibration region (**M**, the set of sites accessible to a species; Figure 1b) using a simulation approach, considering processes of dispersal, colonization, and extinction in the constant current climate and glacial–interglacial climate change frameworks, implemented within the Grinnell package in R (Amaro et al., 2023; Barve et al., 2011; Machado‐Stredel et al., 2021). We kept the default values for this analysis, except kernel spread, which we varied between 0.1 and 5; the simulation period was set up at 65. In light of the limited latitudinal range of this species, we used geographic coordinates for our analyses (WGS, 1984); however, all area calculations were developed in Asia South Albert Equal Area Conic projection. Climatic data were masked to the hypothesized **M** area and a broader area of southwestern Asia for current and future distributions, respectively, using raster extraction routines in ArcGIS 10.3.1.

### 2.4 Ecological niche models and model transfers

The models were developed with the Maxent algorithm using the kuenm R package (Cobos et al., 2019). We tested candidate solutions, including five combinations of the six environmental variables, all 29 possible combinations of the feature types (linear = l, quadratic = q, product = p, threshold = t, and hinge = h), and 18 regularization multiplier settings (0.1, 0.2, 0.3…1.0, 1.25, 1.5, 1.75, 2, 4, 5, 6, 8, and 10). In all, 2610 candidate models were built using all possible combinations of these parameter values. The best set of candidate solutions was selected based on statistical significance (partial ROC, *p* ≤ .05; Peterson et al., 2018), low omission error (omission rates, <5%; Anderson et al., 2003), and a criterion of minimum complexity (Akaike information criterion corrected for small sample sizes, AICc; Warren & Seifert, 2011). In more detail, we evaluated candidate models using partial ROC tests applied to 500 random replicate samples of 50% of the occurrences left out of model calibration (Peterson et al., 2008), and statistical significance was evaluated via a direct count of replicates with AUC ratios ≤1.0. All models were thresholded based on an acceptable calibration omission rate (Peterson et al., 2008) of *E* = 5%, and removed models with omission rates above 0.05. Finally, we filtered models to retain only models with the lowest values of the Akaike information criterion (AICc) (Warren & Seifert, 2011), retaining models with AICc values within one unit of the minimum.

Final models were created using the parameter settings selected, performing 10 bootstrap replicates; we selected the “logistic” output and 10,000 background points in the kuenm package. The final model was transferred across a broad area of southwestern Asia under present‐day conditions for each of the 14 future climate datasets (Wenger & Olden, 2012; Yates et al., 2018), using all three model transfer options: free extrapolation (E), extrapolation and clamping (EC), and no extrapolation (NE).

The median values across replicates were used as the best final estimate of suitability across the region for present‐day conditions. We calculated the range (maximum–minimum) of median values across all GCMs for SSP2‐4.5 and SSP5‐8.5 as a measure of model uncertainty. For projections to future conditions, we calculated the median of replicate medians across all 7 GCMs for SSP2‐4.5 and SSP5‐8.5, and the range among replicates as an index of uncertainty related to the availability of occurrence data. The replicates of final model outputs were thresholded to binary based on an omission error criterion of *E* = 5% (Peterson et al., 2007). We also explored the agreement of changes among the seven GCMs SSP2‐4.5 and SSP5‐8.5 scenarios, and summarized them as stable (the area where is always suitable), gain (range increase of suitable area) and loss (range reduction of suitable area) to represent changes in the same way which shown in geographic projection. Accordingly, we took all projections to future conditions based on distinct GCMs and compared them against the current projection, and quantified the agreement of gain and loss of suitable areas, as well as the stability of suitable and unsuitable conditions (Campbell et al., 2015). All analyses were performed in the kuenm R package (Cobos et al., 2019; R Core Team, 2018) and ArcGIS 10.3.10.

## 3 RESULTS

In all, 72 occurrence data were available for *A. eriophora* from different sources. Three records were omitted from the 72 original datasets for lack of accurate information. Four records were excluded based on the 1.5 km filtering distance and other data‐quality considerations. In all, then, 65 unique occurrences were used for calibration and evaluation of ecological niche models (Table S2). Accessible areas simulated under both changing frameworks of climatic conditions including for most of Iran and small parts of Afghanistan and Pakistan. Figure 1 shows the georeferenced occurrence locations used in this study and the areas identified by our simulations to be accessible to *A. eriophora* over time (**M**) (Figure 1a,b).

Based on high correlations (*r* > .8) and consequent collinearity among climatic variables, nine environmental variables were removed from the analysis. The six variables retained in all five steps of the jackknife procedure were annual mean temperature (bio1), isothermality (bio3), temperature seasonality (bio4), annual precipitation (bio12), precipitation of the driest month (bio14), and precipitation seasonality (bio15). We noted low contributions to model gain by four variables (bio4, bio12, bio14, and bio15) in all four jackknife steps. Accordingly, five sets of environmental variables were explored, including bio1‐bio3; bio1‐bio3‐bio4; bio1‐bio3‐bio4‐bio12; bio1‐bio3‐ bio4‐bio12‐bio14; and bio1‐bio3‐bio4‐bio12‐bio14‐bio15.

We developed 2610 candidate models; 2523 of these models were statistically significantly better than random expectations based on the partial ROC test (*p* < .001; see Table S3); only one model was selected as the best model based on AICc. The best model included two feature types (linear and threshold), with a regularization parameter of one and five environmental variables (bio1‐bio3‐bio4‐bio12‐bio14). This model was explored in terms of implications for potential distribution of *A. eriophora* under present and future conditions.

Based on our results, annual mean temperature (bio1), isothermality (bio3), temperature seasonality (bio4), annual precipitation (bio12), and precipitation in the driest month (bio14) were the influential bioclimatic variables in this study. Bio4 was the most important variable, with 56.2% of the total variable contribution, followed by bio1, bio3, bio14, and bio12 with 22.5%, 12.4%, 7.7%, and 1.1% of the variable contribution, respectively (Figure S1; Table S4).

**FIGURE 2:**
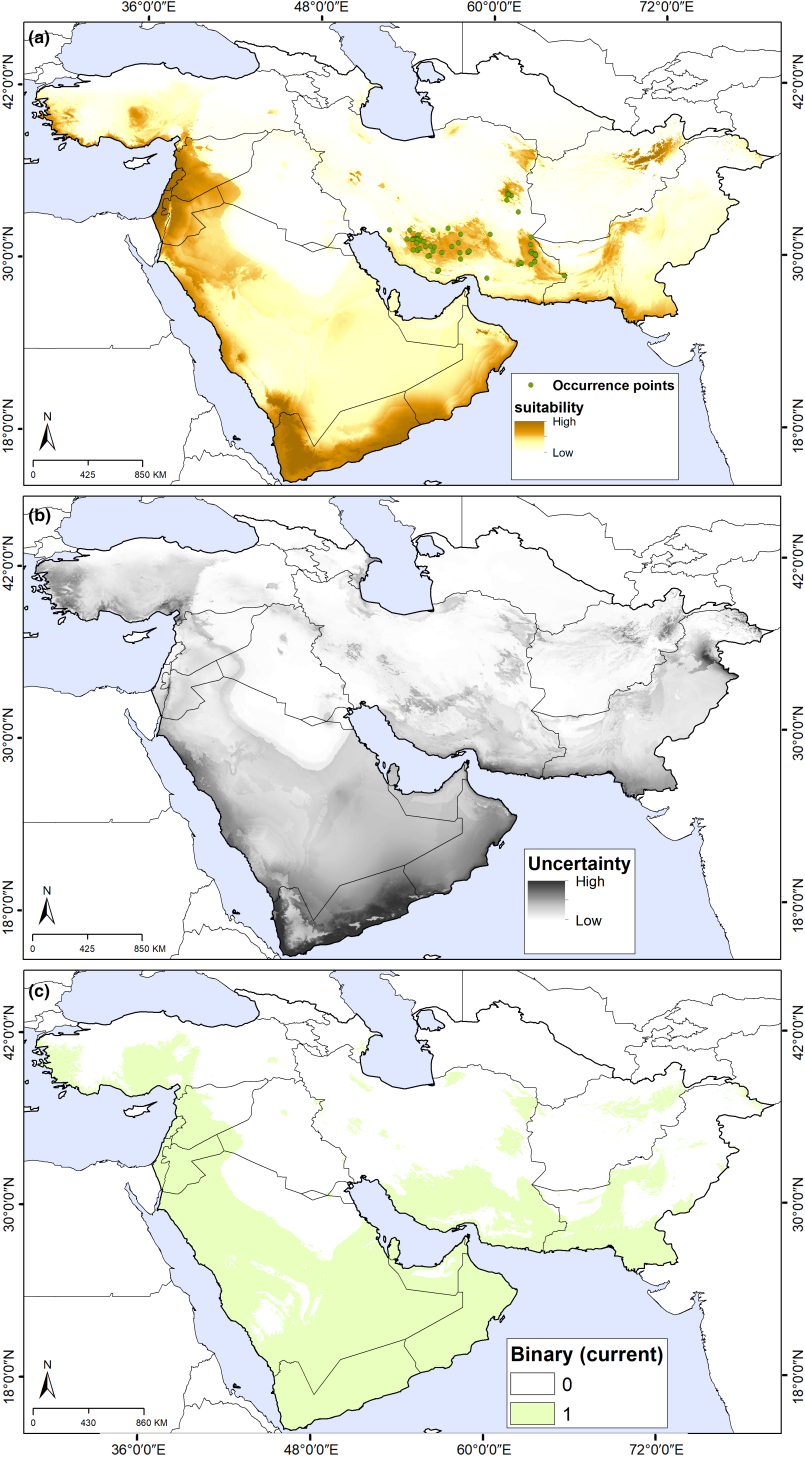
Present‐day suitable areas for *Achillea eriophora* distribution based on Maxent model outputs across southwestern Asia. (a) Predicted areas of high suitability for current conditions (median prediction). (b) The uncertainty associated with predictions of suitability. (c) Binary map based on a least training presence thresholding approach.

The potential distribution under current conditions identified by our models indicated that this species is able to maintain populations in most montane areas, including parts of most countries of southwestern Asia (Figure 2a,c). In Iran, areas of highest suitability extended across the south and east of the country, whereas suitability was markedly lower in the central (desert and low elevation, with warm climatic conditions), and west and north (montane areas with cold climatic conditions) parts of Iran. High suitability was also found in montane areas of Afghanistan and Pakistan, in southern Yemen and Oman, western Saudi Arabia, Syria, and Turkey, as well as throughout Jordan, Israel, and Lebanon. Model uncertainty does not raise serious concerns across much of the study area; however, high levels of uncertainty were observed in southern Yaman, western Saudi Arabia, Turkey, and eastern Pakistan (Figure 2b).

**FIGURE 3:**
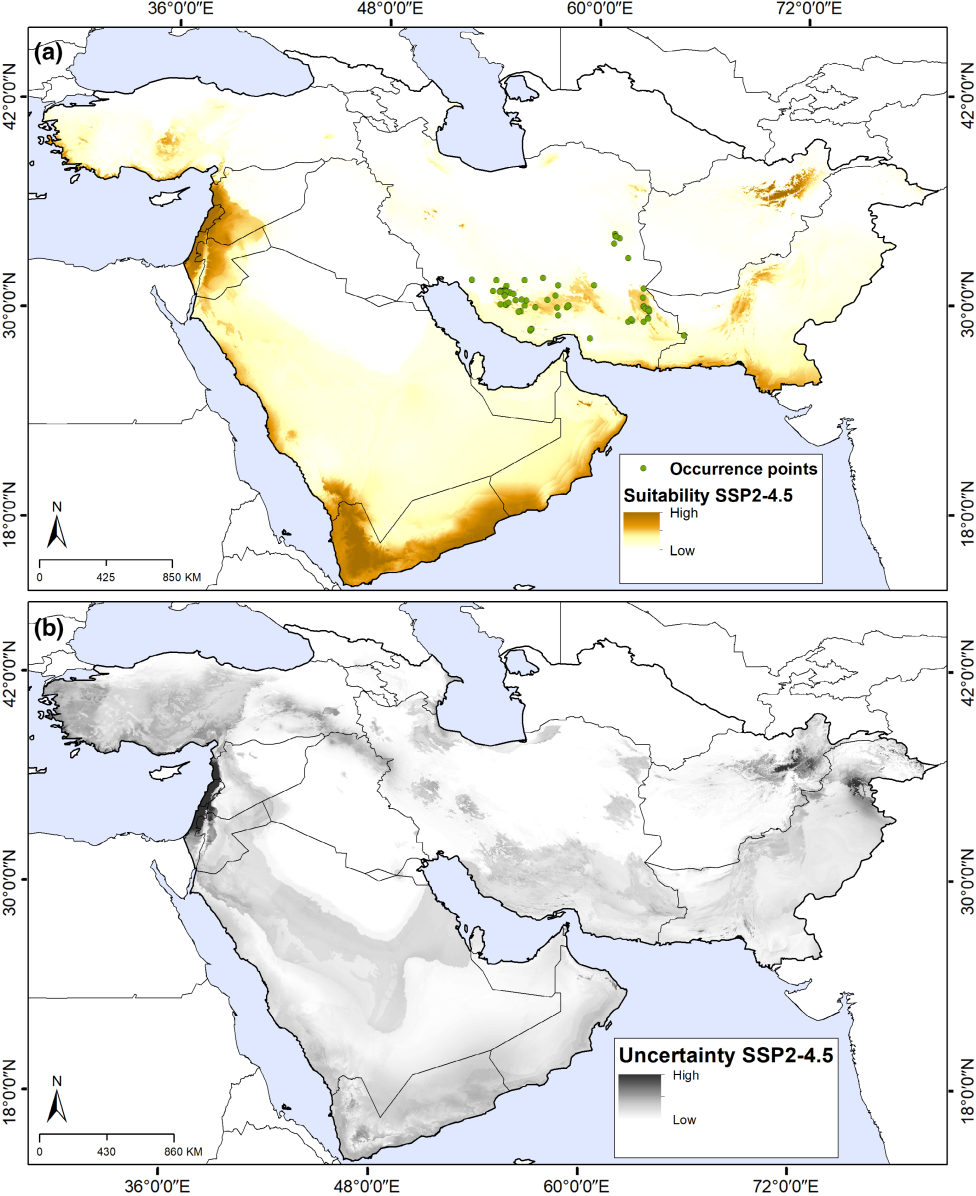
Future suitable areas for *Achillea eriophora* distribution based on Maxent model outputs under Shared Socioeconomic Pathways (SSP2‐4.5) across southwestern Asia. (a) Predicted areas of high suitability for future conditions (median prediction). (b) The uncertainty associated with predictions of suitability.

**FIGURE 4:**
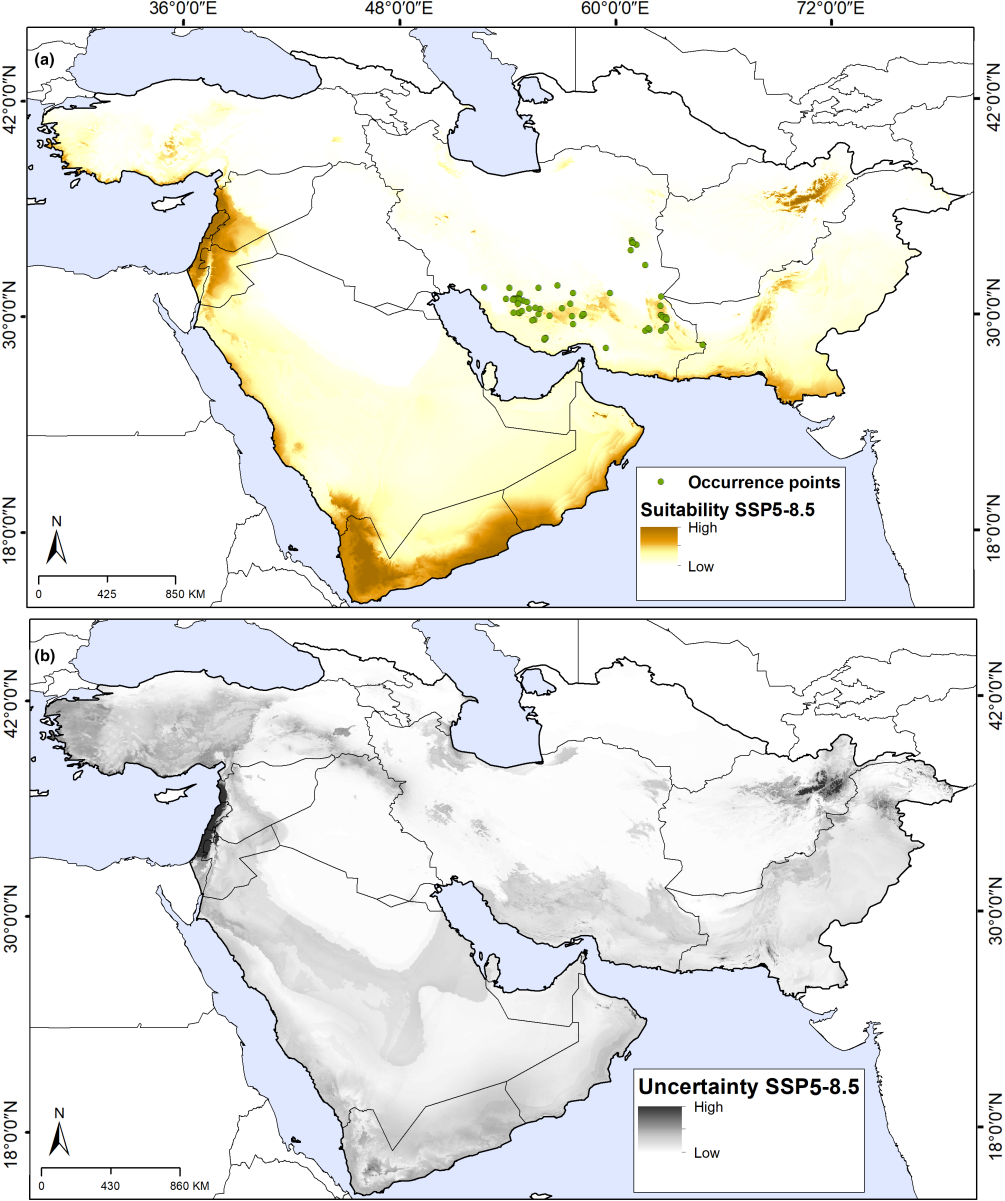
Future suitable areas for *Achillea eriophora* distribution based on Maxent model outputs under shared socioeconomic pathways (SSP5‐8.5) across southwestern Asia. (a) Predicted areas of high suitability for future conditions (median prediction). (b) The uncertainty associated with predictions of suitability.

Model transfers revealed an overall distributional pattern similar to that under present‐day conditions under both SSP2‐4.5 and SSP5‐8.5 scenarios (Figures 3a and 4a; Figures S2a and S2b). Based on these scenarios, suitable areas expanded generally, and shifted toward higher elevations and northward in Iran, Turkey, Afghanistan, and Pakistan. We noted increases in suitability with low uncertainty under both scenarios (SSP2‐4.5 and SSP5‐8.5); some areas of eastern Afghanistan and Pakistan, and most of Israel and Lebanon (Figures 3b and 4b).

Based on a composite map of current and future scenarios (Figure 4a,b), areas of range reduction (loss) were predicted in the south and scattered areas of Iran, Turkey, Pakistan, eastern Afghanistan, eastern Syria, eastern Jordan, and central Saudi Arabia. Range expansion (gain) was anticipated in western Turkey, montane areas (Zagros Mountains), southern Iran, central Afghanistan, central Pakistan, and central Saudi Arabia. In general, the models identified a future potential distribution that reflected only a minimal expansion of the distributional potential of *A. eriophora* from the present distribution. Accordingly, the potential distribution of *A. eriophora* increased by 7.5% and 9.2% from current conditions to SSP2‐4.5 and SSP5‐8.5 conditions, respectively.

## 4 DISCUSSION

Ecological niche modeling is a widely used tool in various fields of plant ecology to predict the habitat suitability of endemic, medicinal, and endangered species (Karami et al., 2022; Velazco et al., 2017). Based on a Global Bioclimatic Classification (Djamali et al., 2011), *A. eriophora* is distributed in six bioclimatic zones, including (1) Mediterranean desertic‐continental (Mdc), with low annual precipitation and extended summer drought; (2) Mediterranean xeric‐continental (Mxc), with summer drought and low total amount of annual precipitation; (3) Mediterranean pluviseasonal‐continental (Mpc), with high winter precipitation; (4) Mediterranean xeric‐oceanic (Mxo), with long summer drought, low annual precipitation, and high average winter temperature minima; (5) Tropical desertic (Trd), with a peak summer rainfall in eastern Iran in July; and (6) Tropical xeric (Trx), with most annual precipitation in winter (Djamali et al., 2011). Our results corroborated this summary within the study area, including the influences of annual precipitation (bio12), precipitation in the driest month (bio14), and temperature seasonality (bio4) on the geographic potential of the species. Low annual precipitation and summer drought have apparently affected morphological characteristics of *A. eriophora* (Djamali et al., 2011), e.g., in developing dense woolly hairs, many woody branches, small leaf size, leaf rolling, and stomatal positions as crypts (Azani et al., 2009; Mozaffarian, 2008; Seleiman et al., 2021).

Based on our results, when precipitation in the driest month (bio4) is 5°C, suitability for *A. eriophora* is high (>0.9); it then decreases with the continuous increase in precipitation in the driest month (bio4) to <0.1 when precipitation in the driest month (bio4) = 9°C. When annual mean temperature (bio1) is 2°C, suitability for *A. eriophora* is 0.8; suitability decreases lightly with the continuous increase in annual mean temperature (bio1). Suitability is close to 0 with annual mean temperature (bio1) values above 28°C (Figure S1). Bioclimatic parameters, especially temperature in mountain habitats, have been highlighted as an important parameter in previous studies (Robiansyah, 2018). The importance of annual precipitation (bio12) was also confirmed for two endemic plant species in the Irano‐Turanian region (Behroozian et al., 2020; Karami et al., 2022).

Suitable habitats for *A. eriophora* were identified in mountainous areas in most countries of southwest Asia under current conditions. However, successful colonization by species in disjunct suitable areas depends on the dispersal abilities of the species. Knowledge of dispersal abilities of the species is limited, although previous studies on the related species *A. millefolium* indicate that this species can regenerate branches that reach up to 30 cm deep in disturbed soils through rhizome fragments, which can form new plants at the top of the rhizome in intact soil. *Achillea millefolium* seeds also can disperse up to 2 m by wind (Aleksoff, 1999; Bork et al., 1996; Kuntz, 1982; Stickney, 1989). Given these mechanisms, it seems unlikely that *A. eriophora* would be able to colonize the most distant areas, regardless of their suitability.

**FIGURE 5:**
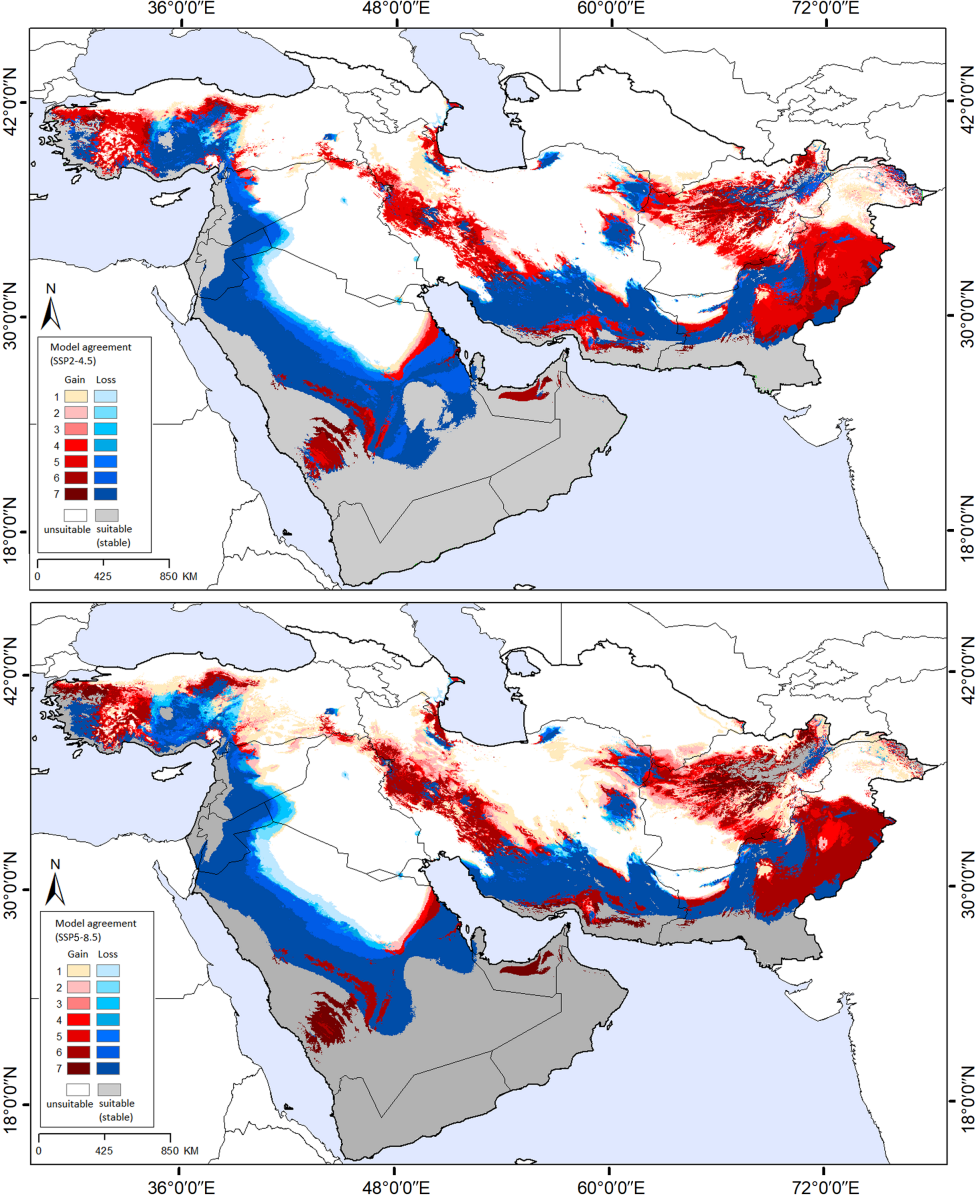
Predicted suitable areas and changes in suitability of *Achillea eriophora* based on Maxent model outputs under climate change scenarios SSP2‐4.5 and SSP5‐8.5 and agreement between different general circulation models for the study area.

Transfers of the model to future climate scenarios (SSP2‐4.5 and SSp5‐8.5) found much the same distributional pattern as under current conditions. However, a slight increase was observed in the suitable habitats under the future conditions compared to the current conditions; with the transfer of the model under the SSP2‐4.5 and SSp5‐8.5 scenarios (7.5% and 9.2%). The only difference was that the border areas of southern and western Saudi Arabia and southern Yemen and Oman, which do not presently hold suitable conditions for the species, become suitable habitats. More generally, the species' range is anticipated to be almost entirely restricted to montane areas: high‐suitability habitats expand and shift toward higher elevations and northward (Figures 3a, 4a, 5; Figure S2a,b). Dispersal is a crucial factor in shifting species' ranges, particularly along elevational gradients (Normand et al., 2009). As maintained above, this species disperses its seeds only over relatively short distances, and so will likely not be able to colonize potentially suitable regions across the study area.

However, on more local and more clearly accessible scales, *A. eriophora* will experience relatively high losses of suitability in its actual habitats under future climate scenarios (Figures  5). These losses on local and regional scales for *A. eriophora* are considerably higher than what has been found for other species in this region (Behroozian et al., 2020; Karami et al., 2022). For example, Karami et al., (2022) reported only minimal loss in habitats of *Nepeta glomerulosa* in southern and central Asia under future scenarios. Behroozian et al., (2020) found changing and shifting suitability patterns for an endemic plant species on more local scales under both current and future conditions. Hence, climate change can be considered a serious threat to the species in the future. Other ecological factors also play important roles in shaping species' distributions, such as biotic interactions and anthropogenic disturbances (Escobar et al., 2015; Wisz et al., 2013). Particularly, the negative effects of anthropogenic activities, such as irregular harvesting, and development can fragment species' habitats and reduce the distribution of the species in the future (Mohammadi et al., 2021, 2023).

## 5 CONCLUSION

The results of the present study explored the potential impacts of current and future climate on the distribution of *A. eriophora*. The study revealed that impacts of climate change on the distributional potential of the spices will be minimal. However, *A. eriophora* is under threat of extinction at local and accessible scales owing to relatively high losses of suitability in its actual habitats under future climate scenarios. Furthermore, the models presented here predict that high suitable habitats expand and shift towards higher elevations and northward; such that climate change particularly threatens species if it is not able to colonize new regions. Hence, further detailed study is needed to evaluate the dispersal abilities, as they shape the species' conservation status, and provide reference for the future protection and management of *A. eriophora* in the study area. Specifically, anthropogenic activities such as irregular harvesting exacerbate the negative effects of climate changes in habitats of the species. This study not only provides valuable information on likely impacts of climate change on *A. eriophora* but may also help in developing basic information for conservation and management of endemic species in arid and semi‐arid regions. Here, we propose approaches for conservation of this species, including *in situ* and *ex situ* conservation, and conventional seed bank storage.

## AUTHOR CONTRIBUTIONS

**Fariba Noedoost:** Conceptualization (supporting); data curation (lead); project administration (supporting); resources (lead); visualization (lead); writing – original draft (supporting); writing – review and editing (equal). **Maryam Behroozian:** Conceptualization (lead); formal analysis (lead); investigation (lead); methodology (lead); validation (lead); writing – original draft (lead); writing – review and editing (equal). **Sahar Karami:** Data curation (equal); resources (equal); visualization (equal); writing – original draft (supporting); writing – review and editing (equal). **Mohammad Reza Joharchi:** Data curation (equal); resources (equal); writing – review and editing (equal).

## CONFLICT OF INTEREST STATEMENT

The authors declare no competing interests.

## Supporting information


Appendix S1.

## Data Availability

The data that support the findings of this study are available in the Supplementary Material of this article.
