# Supplementary material for: Potential impacts of climate change on the geographic distribution of Achillea eriophora DC., a medicinal species endemic to Iran in southwestern Asia
Source: Ecol Evol. 2024 Apr 25;14(4):e11241. doi: 10.1002/ece3.11241 (PMC11045919; doi:10.1002/ece3.11241)
Supplement: Supplementary file 1 — Appendix S1. [file ECE3-14-e11241-s001.zip › ece311241-sup-0001-AppendixS1.docx]

**Supplementary**

**Potential impacts of climate change on the geographic distribution of *Achillea eriophora*, a medicinal plant species in southwestern Asia**

**Fariba Noedoost ^1^, Maryam Behroozian^2^*, Sahar Karami^3^, Mohammad Reza Joharchi^2^**

Corresponding author:

E–mail: maryam.behroozian94@gmail.com

Address: Herbarium FUMH, Ferdowsi University of Mashhad, Mashhad, Iran.

**Table S1.** A list of models used for SSP2-4.5 and SSP2-8.5 scenarios.

| **No.** | **Model name** | **Abbreviation name** |
| --- | --- | --- |
| **1** | Beijing Climate Center (BCC) Climate System Model | BCC-CSM2-MR |
| **2** | Australian Community Climate and Earth System Simulator Climate Model Version 2 | CCESS-CM2 |
| **3** | Centro Euro-Mediterraneo sui Cambiamenti Climatici - Earth System Model Version 2 | CMCC-ESM2 |
| **4** | Goddard Institute for Space Studies Earth System Model | GISS-E2-1-G |
| **5** | Hadley Centre Global Environment Model in the Global Coupled Configuration 3.1 | HadGEM3-GC31-LL |
| **6** | Institut Pierre-Simon Laplace- Coupled Model Intercomparison Project | IPSL-CM6A-LR |
| **7** | Model for Interdisciplinary Research on Climate Version 6 | MIROC6 |

**Table S2.** Summary of occurrence data used in this study

| Area | Latitude | Longitude | Elevation | Herbarium number | Herbarium name |
| --- | --- | --- | --- | --- | --- |
|  |  |  |  |  |  |
| Yazd: about 30 km southwest of Merost, Bagh Shadi (BUI) | 53.9474 | 30.29094 | 2148 | 82954 | TARI |
| Fars: Shiraz, Park Bamo | 53.08652 | 29.57984 | 1740 | 71473 | TARI |
| Fars: Firouzabad, Meimand, Sepidar : Mount from Tang Riz | 52.88218 | 28.89287 | 2033 | 71450 | TARI |
| Fars | 52.84178 | 28.90208 | 2256 |  | TARI |
| Fars: 13 km from Zarghan, towards Band Amir | 52.82078 | 29.78806 | 1588 | 66927 | TARI |
| Fars: between Persepolis and Naqsh Rostam (XP4) | 52.89283 | 29.97566 | 1633 | 83003 | TARI |
| Fars: 15 to 20 km from Shiraz to Isfahan (XN1) | 52.55702 | 29.70298 | 1827 | 82993 | TARI |
| Fars: mountains north of Shiraz (XNI) | 52.68187 | 29.68187 | 2000 | 46608 | TARI |
| Fars: 33 km from Lar to Bestak, mountains south-east of Hermod village | 54.34541 | 27.49098 | 834 | 41834 | TARI |
| Fars: Bamo Mount | 52.62285 | 29.67617 | 2471 | 24568 | TARI |
| Fars: 80 km southeast of Shiraz | 53.00479 | 29.00857 | 1430 | 6253 | TARI |
| Fars: 13 kilometers east of Jahrom | 53.70453 | 28.50963 | 1253 | 6346 | TARI |
| Fars: 32 kilometers west of Shiraz | 52.12196 | 29.67887 | 1980 | 8304 | TARI |
| Fars: between Sarvostan and Fasa, 20 km before the research station in the forest | 53.42108 | 29.14532 | 1700 | 69312 | TARI |
| Fars: 21 km from Shiraz to Sarvostan, southwest of Maharlo lake (NX3) | 52.77751 | 29.41992 | 1350 | 46692 | TARI |
| Fars: Firozabad, 5 km from Firozabad to Kavar | 52.54672 | 28.89966 | 1350 | 71414 | TARI |
| Fars: 25 km southeast of Fasa, Shalo village, Rose Mountain (XN4) | 53.93736 | 28.82151 | 1830 | 46791 | TARI |
| Fars: south of Estehbanat, Bash Mountain (BT2) | 54.04169 | 29.11122 | 1932 | 47007 | TARI |
| Fars: Fasa, Roniz, Marghak Valley (YN4) | 53.7616 | 29.17842 | 1588 | 46955 | TARI |
| Fars: Hossein Abad Protected Area | 52.30613 | 30.30746 | 1850 | 46675 | TARI |
| Fars: Bamo Park, left valley | 52.83354 | 29.69881 | 1750 | 17578 | TARI |
| Fars: Bamo Park, Tang Chah Mahki | 52.74231 | 29.68774 | 1800 | 17715 | TARI |
| Fars: Kherameh | 53.29611 | 29.50722 | 1602 | 47092 | HSHU |
| Fars prov. Shiraz, slopes 1-1.5 km E. Gahvarch-Deed, just N. of the town | 52.6625 | 29.64056 | 1890 | 716 | S |
| Fars: Lar. Iranian Haji Abad near Tarum | 55.88317 | 28.25786 | 900 | 3491 | NMNH |
| Fars: Bamo Mountain, near Shiraz | 52.87629 | 29.64341 | 2134 | 739K | E |
| Fars: Perspolis, in the hills to the Salt Lake (Namek-Derja) | 52.79247 | 29.40207 | 1561 | 0056451 | W |
| Fars: Shiraz mountains | 52.51722 | 29.64778 | 1600 | 1437653 | H |
| Fars | 54.55989 | 28.72558 | 1165 | G-DC-306826/1 | G |
| Fars Jahrom | 53.58679 | 28.48631 | 1176 | G-G-213038/1 | G |
| Kerman: 32 km towards Anar from Babak city (CUI) | 55.00287 | 30.42449 | 2270 | 56455 | TARI |
| Kerman: near Sarcheshme (CU4, DU2) | 55.87355 | 30.00046 | 2450 | 56408 | TARI |
| Kerman: about 20 kilometers from Khabr to Koht | 56.38592 | 28.7316 | 2200 | 25137 | TARI |
| Kerman: Khabr and Rochun protected area, 50 km southwest of Baft, east of Khbar mountain | 56.47251 | 28.82831 | 2850 | 1709 | TARI |
| Kerman: 50 km after Sirjan to Neyriz | 55.26278 | 29.15417 | 1788 | 47093 | HSHU |
| Kerman: Khabr va Rouchun protected region, 50 km SSW of Baft. E side Kuh-e Khabr, 12 km E of Khabr | 56.43333 | 28.83333 | 2850 | 1707 | E |
| Kerman: Sirjan | 55.73108 | 29.41098 | 1741 | 1710 | E |
| Kerman: Jopar | 57.95978 | 30.01488 | 2149 | 56450 | HSHU |
| Khuzestan: 68 kilometers east of Behbahan | 50.90635 | 30.31831 | 739 | 885 | TARI |
| Hormozgan: 73 kilometers from Lar to Bastak | 54.28027 | 27.39101 | 500 | 41855 | TARI |
| Hormozgan: about 50 kilometers from Hajiabad to Sirjan, between Baghat and Chaghok well | 55.81751 | 28.70933 | 1600 | 52608 | TARI |
| Hormozgan: Lar, Hormood, Kuh-e-Pardis, 12 km after the beginning of Kuh-e-Pardis | 54.28556 | 27.47139 | 1500 | 47091 | HSHU |
| Balochistan: about 40 km north of Khash, Torsh ab | 61.13065 | 28.48898 | 1900 | 22909 | TARI |
| Balochistan: Bazman, between Iranshahr and Bam, Khezr mountain | 60.1589 | 27.94503 | 1500 | 23154 | TARI |
| Baluchistan: 20 km from Khash towards Iranshahr | 61.07616 | 28.10671 | 1450 | 22951 | TARI |
| Balochistan: Bazman Mountain, between Iranshahr and Bam | 59.92582 | 27.9056 | 1203 | 23080 | TARI |
| Balochistan: 85 to 100 km from Khash towards Bazman | 60.81815 | 27.90343 | 1600 | 22989 | TARI |
| Baluchistan: 65 km from Khash to Zahedan, Murtek | 60.79829 | 28.77441 | 2100 | 53400 | TARI |
| Balochistan: Taftan mountains, Chukalki between Jun Abad and Kharistan | 60.83002 | 28.77428 | 2200 | 52957 | TARI |
| Balochistan: Zahedan: the pass between Junabad and Kharistan (about 42 km from Khash-Zahedan Road) | 60.91551 | 28.65113 | 2200 | 52931 | TARI |
| Balochistan: 30 km before Zahedan from Zabul road | 60.82388 | 29.82227 | 1200 | 1094 | TARI |
| Balochistan: 18 km south of Zahedan | 60.77624 | 29.29452 | 1700 | 9407 | TARI |
| Baluchistan: Eastern slopes of Kuh-e-Bazman | 60.1 | 28.03333 | 1700 | 00582439  75.468 | E |
| Baluchistan: Zaboli to 11km West of Kuhuk | 63.13571 | 27.10517 | 1150 | 75.519  E00582440 | E |
| Balochistan: between Zahedan (Dozdab) and Khash (Vasht, Kavash) | 61.06823 | 28.06823 | 1450 | 4264 | NMNH |
| Sistan and Baluchistan: Mount Taftan | 61.08591 | 28.65448 | 2512 | 52959 | TARI |
| Baluchistan: on the southern rocky slopes of Taftan volcano near Torshab | 61.11852 | 28.55201 | 2600 | 54829 | S |
| Khorasan: Birjand, 5 km from Mod, Qala mountains | 59.45085 | 32.72637 | 2000 | 17683 | FUMH |
| Khorasan: Birjand, Amin abad | 59.16841 | 32.96913 | 1834 | 30348 | FUMH |
| Khorasan: south of Birjand, Baghran Mountains, Band Dareh | 59.21833 | 32.81522 | 1740 | 44937 | FUMH |
| Khorasan: Birjand, Omarshah Band mountains | 59.18481 | 32.82982 | 1684 | 34259 | FUMH |
| Khorasan: Birjand, northwest of Nehbandan, 6 km from Nehbandan to Gadamgah | 59.92532 | 31.56989 | 1350 | 28340 | FUMH |
| Khorasan: Birjand, Sarchah, Mazar Shah Suleiman Ali (Agha Seyed Ali) | 59.1198 | 32.4124 | 1900 | 30080 | FUMH |
| Hormozgan: about 50 km north-east of Sendark | 57.71722 | 26.92056 | 1115 | 459 | COI |
| Khorasan: mountains 4 km south of Birjand | 59.2325 | 32.82611 | 1620 | P04381502 | P |

Royal Botanic Garden Edinburgh Herbarium (E)

Swedish Museum of Natural History (S)

Herbarium of the University of Coimbra (COI)

Geneva Herbarium – General Collection (G)

Museum Botanicum University Helsinki (H)

(P) at the Herbarium of the Muséum national d'Histoire Naturelle (MNHN - Paris)

herbarium of the Museum of Natural History in Vienna, Austria (W)

Herbarium of Shiraz University (HSHU)

**Table S3.** Summary of model parameter settings explored and tested in this study

| Variable | Percent contribution | Permutation importance |
| --- | --- | --- |
| bio4 | 56.2 | 56.9 |
| bio1 | 22.5 | 18.4 |
| bio3 | 12.4 | 5.5 |
| bio14 | 7.7 | 17.2 |
| Bio12 | 1.1 | 2.1 |

**Table S4.** Analysis of variables contributions used in habitat suitability *Achillea eriophora.*

(excel file)


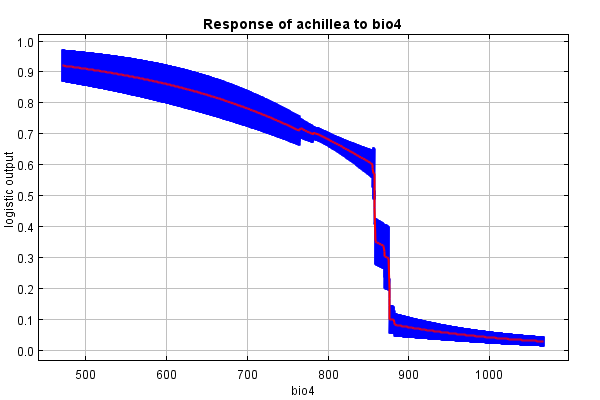

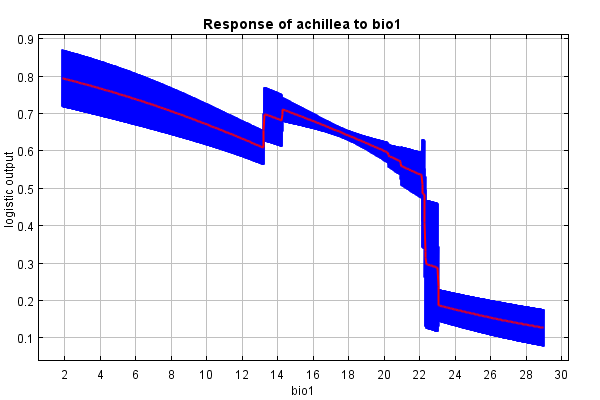


**Figure S1.** The major predictors of suitability habitats of *Achillea eriophora*

**
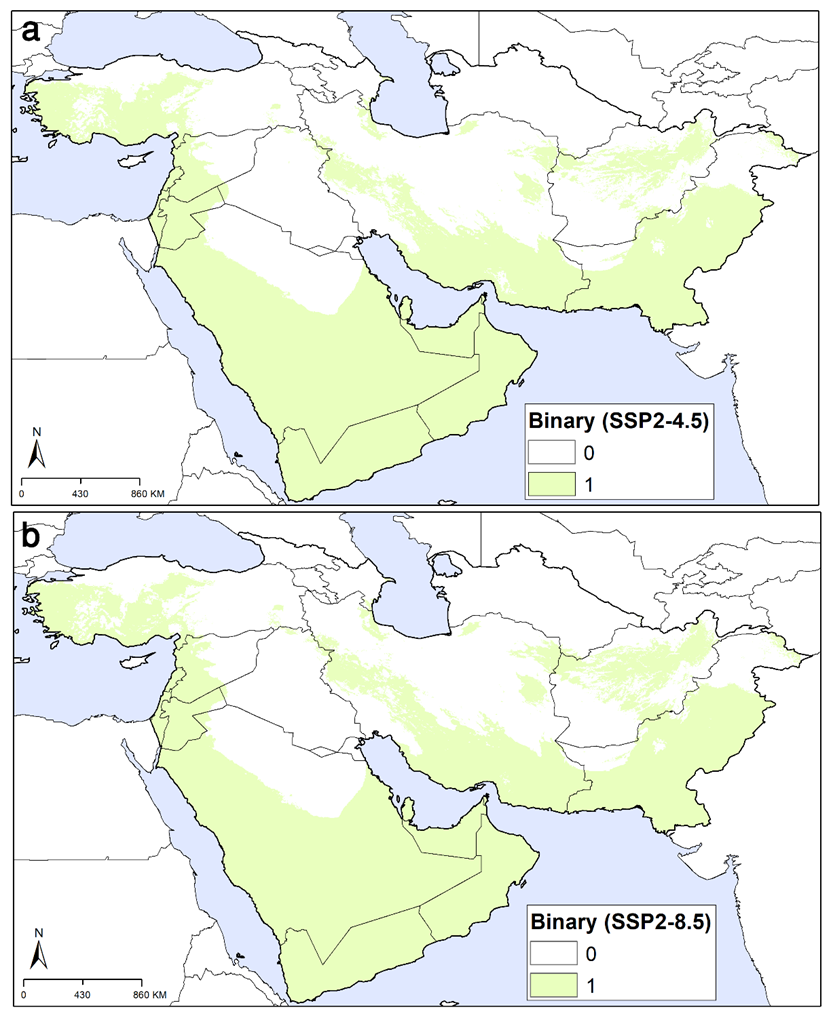
**

**Figure S2:** The preceding map turned to a binary prediction based on a modified least training presence thresholding approach (*E* = 5%) under two scenarios of SSP2-4.5 and SSP2-8.5.
