# Supplementary figures and images for: Potential impacts of climate change on the geographic distribution of Achillea eriophora DC., a medicinal species endemic to Iran in southwestern Asia
Source: Ecol Evol. 2024 Apr 25;14(4):e11241. doi: 10.1002/ece3.11241 (PMC11045919; doi:10.1002/ece3.11241)

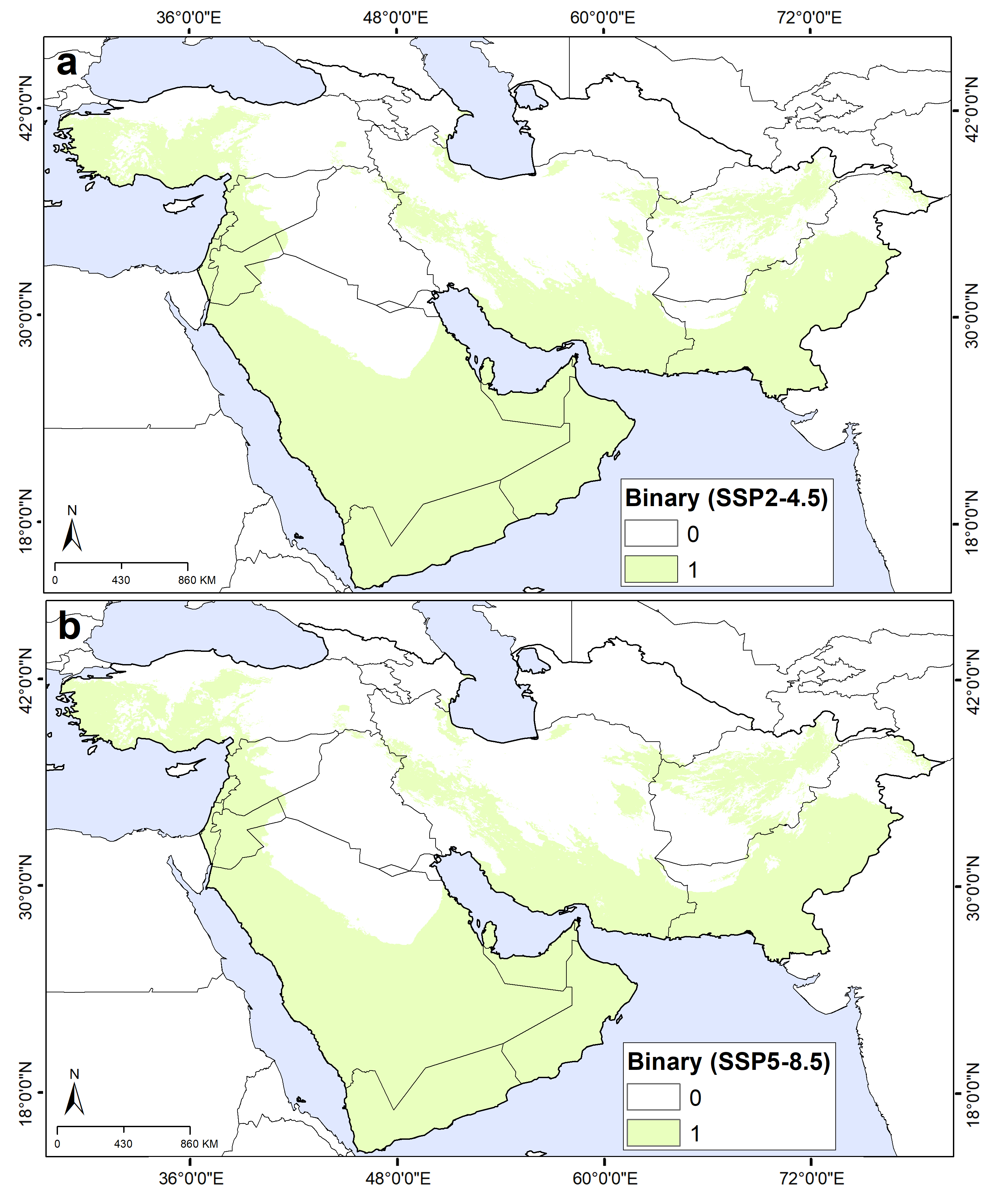

Supplement: Supplementary file 1 — Appendix S1. [file ECE3-14-e11241-s001.zip › Figure S2.tif]
